# Quality of care for secondary cardiovascular disease prevention in 2009–2017: population-wide cohort study of antiplatelet therapy use in Scotland

**DOI:** 10.1136/bmjqs-2023-016520

**Published:** 2023-09-29

**Authors:** Inna Thalmann, David Preiss, Iryna Schlackow, Alastair Gray, Borislava Mihaylova

**Affiliations:** 1 Health Economics Research Centre, Nuffield Department of Population Health, University of Oxford, Oxford, UK; 2 MRC Population Health Research Unit, Clinical Trial Service Unit & Epidemiological Studies Unit, Nuffield Department of Population Health, University of Oxford, Oxford, UK; 3 Health Economics and Policy Research Unit, Wolfson Institute of Population Health, Queen Mary University of London, London, UK

**Keywords:** chronic disease management, clinical practice guidelines, general practice, healthcare quality improvement, health policy

## Abstract

**Background:**

Antiplatelet therapy (APT) can substantially reduce the risk of further vascular events in individuals with established atherosclerotic cardiovascular disease (ASCVD). However, knowledge regarding the extent and determinants of APT use is limited.

**Objectives:**

Estimate the extent and identify patient groups at risk of suboptimal APT use at different stages of the treatment pathway.

**Methods:**

Retrospective cohort study using linked NHS Scotland administrative data of all adults hospitalised for an acute ASCVD event (n=150 728) from 2009 to 2017. Proportions of patients initiating, adhering to, discontinuing and re-initiating APT were calculated overall and separately for myocardial infarction (MI), ischaemic stroke and peripheral arterial disease (PAD). Multivariable logistic regression and Cox proportional hazards models were used to assess the contribution of patient characteristics in initiating and discontinuing APT.

**Results:**

Of patients hospitalised with ASCVD, 84% initiated APT: 94% following an MI, 83% following an ischaemic stroke and 68% following a PAD event. Characteristics associated with lower odds of initiation included female sex (22% less likely than men), age below 50 years or above 70 years (aged <50 years 26% less likely, and aged 70–79, 80–89 and ≥90 years 21%, 39% and 51% less likely, respectively, than those aged 60–69 years) and history of mental health-related hospitalisation (45% less likely). Of all APT-treated individuals, 22% discontinued treatment. Characteristics associated with discontinuation were similar to those related to non-initiation.

**Conclusions:**

APT use remains suboptimal for the secondary prevention of ASCVD, particularly among women and older patients, and following ischaemic stroke and PAD hospitalisations.

**WHAT IS ALREADY KNOWN ON THIS TOPIC**

**WHAT THIS STUDY ADDS**

- This national study in Scotland provides new insights into the full extent of suboptimal mono-antiplatelet therapy and dual-antiplatelet therapy use across different populations and disease types in the treatment pathway following hospitalisation for ASCVD.
- Only 84% of patients initiated APT after hospitalisation for ASCVD of whom 22% later discontinued therapy, with systematic APT undertreatment observed among patients with peripheral arterial disease and stroke, as well as women and patients aged <50 years or ≥70 years.

**HOW THIS STUDY MIGHT AFFECT RESEARCH, PRACTICE OR POLICY**

- Improvements to APT use in patients with ASCVD would provide substantial benefits to this population by preventing as many as 5% of subsequent ASCVD events.
- Women and older patients, as well as patients who suffer ischaemic stroke and peripheral arterial disease events, remain particularly undertreated and require further focus.

Antiplatelet therapies (APT) have been shown to reduce cardiovascular morbidity and mortality in patients with established atherosclerotic cardiovascular disease (ASCVD).^1^,^4^ Given their efficacy and favourable safety and cost-effectiveness profiles, national clinical guidelines, such as those issued by the National Institute for Health and Care Excellence for England and Wales and the Scottish Intercollegiate Guidelines Network Consortium, have recommended the long-term use of APT for the secondary prevention of ASCVD.

Few studies have analysed the adequacy of APT use for secondary prevention of ASCVD. Despite guideline recommendations, studies found suboptimal APT utilisation.^5^,^7^ However, information on the extent of APT use at different treatment stages, and the role of particular individual characteristics, is very limited. Policy makers and providers would benefit from a detailed understanding of APT use at different treatment stages to inform development of quality-improvement interventions and policies. Such evidence can be generated from reliable population-wide individual patient data.

This study sought to estimate the extent of suboptimal APT use for the secondary prevention of ASCVD in Scotland, overall and across subgroups by age, sex, types of ASCVD and other characteristics. We examined associations between patient characteristics at different stages of the treatment pathway to identify patient groups at increased risk of suboptimal medication use.

## Methods

### Data

Details of the study methods and data have been described previously in an analysis of statin therapy use for the secondary prevention of CVD in Scotland.^8^ Briefly, the National Health Service (NHS) Scotland provides free healthcare (including free prescription medications since April 2011) at the point of use to all individuals living in Scotland. This retrospective open cohort study used population-wide individual patient data for all individuals hospitalised for ASCVD comprising four linked and anonymised routine healthcare datasets: (1) hospital admissions, (2) specialty mental health admissions, (3) national death records and (4) the Prescribing Information System (PIS), which contained dispensing information for individuals. Individuals were followed between 1 October 2009 and 31 December 2017 (online supplemental table A and online supplemental figure A).

### Study population

All Scottish residents aged 18 years or older were included if they had a main discharge diagnosis for an ASCVD event between 1 October 2009 and 3 July 2017, and therefore should have been offered guideline-recommended APT for the secondary prevention of ASCVD.^9^,^11^

ASCVD events were categorised into myocardial infarction (MI) and ischaemic stroke based on the International Classification of Diseases 10th Revision (ICD-10) codes and minimum hospital length of stay (LOS) of 1 day, peripheral arterial disease (PAD) based on ICD-10 codes and other ASCVD based on ICD-10 codes (including diagnostic codes for MI and ischaemic stroke with a LOS of <1 day) (online supplemental table B). In accordance with clinical guidelines, patients with atrial fibrillation are likely to be prescribed anticoagulation therapy instead of APT, and were therefore excluded based on ICD-10 codes at index admission (online supplemental table B). Additionally, patients with ASCVD who underwent a percutaneous coronary intervention (PCI) with and without stenting during hospitalisation, and thus should have been offered guideline-recommended dual-antiplatelet therapy (DAPT) to reduce risks of subsequent vascular events,^9^,^11^ were identified.

Following exclusion criteria (non-Scottish residents, emigration and death within 150 days after discharge, hospital LOS of >90 days, medication supply for >365 days and medications in non-tablet/non-capsule format; online supplemental figure B), the final study population size was 150 728 individuals. Individuals were followed for up to 8 years (average 4.6 years) from their index ASCVD event (recorded on/after 1 October 2009) until study end (ie, 31 December 2017), emigration or death, depending on which event occurred first.

### Primary outcomes: antiplatelet therapy initiation, adherence, discontinuation and re-initiation

**Figure 1:**
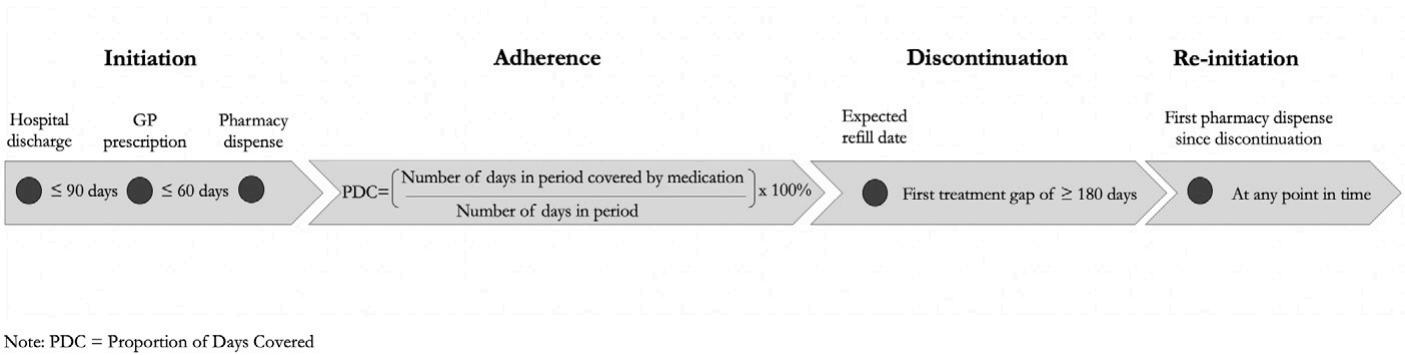
Schematic of the outcome measures initiation, adherence, discontinuation and re-initiation. GP, general practitioner; PDC, proportion of days covered.

APT use (ie, mono-antiplatelet therapy or DAPT combinations of aspirin, clopidogrel, ticagrelor, prasugrel and dipyridamole; online supplemental table C) was assessed at four stages of the patient treatment journey: APT initiation, adherence, discontinuation and re-initiation (figure 1). APT initiation was defined as individuals being prescribed APT within 90 days from index discharge and dispensed within 60 days from that prescription, irrespective of their APT usage status prior to the hospitalisation. Adherence was defined as the degree to which patients follow the agreed prescription instructions from a healthcare provider.^12^ Patients’ prescription records were used to indirectly measure the proportion of days covered (PDC), which was calculated as the ratio of the number of days the patient is covered by the medication in a period to the total number of days in the period.^13^ Adherence was defined as a PDC threshold of ≥80%, and was measured from the date an individual initiated treatment until study end, or date of discontinuation, emigration or death, depending on which event occurred first. Discontinuation was measured as the start of the first continuous medication treatment gap of 180 days or more since initiation to ensure that individuals with a medication supply of 90–180 days (0.5% of individuals) were not erroneously excluded. A binary outcome measure was created to indicate if a patient (1) discontinued or (2) did not discontinue treatment at any point in time after treatment initiation. Re-initiation was defined as a record of having been dispensed APT at any point in time after the first 180-day treatment gap.

### Patient characteristics

The following characteristics were assessed: sex, age at index event date, socioeconomic deprivation quintile, number of comorbidities, previous mental health hospitalisation, history of previous ASCVD event and/or previous APT use and discharge calendar year (online supplemental tables D and E).

### Statistical analysis

The proportions of individuals who did not initiate therapy, were not adherent, discontinued treatment and did not re-initiate therapy were calculated. Multivariable logistic regression models were used to study the association between patient characteristics and discharge calendar year and the likelihood of initiating APT after an ASCVD event, and the likelihood of initiating DAPT versus antiplatelet monotherapy among individuals who underwent PCI. For individuals who initiated APT, a Cox proportional hazards model^14^ was used to assess the role of patient characteristics in the discontinuation of APT, censored for death and emigration. A Schoenfeld residuals test showed that the proportional hazards assumptions were met.^15^

## Results

### Antiplatelet therapy initiation

Between 2009 and 2017, 196 984 individuals were hospitalised for ASCVD. Following exclusion criteria (online supplemental figure B), the final study population included 150 728 individuals. Of these individuals, 34 257 (23%) died at some point in time between their index discharge and the study end date (online supplemental table E). Demographic characteristics at discharge by initiation and discontinuation status are presented in table 1 and online supplemental table E, and across ASCVD types in online supplemental tables F and G.

**Table 1:** Participant characteristics of patients who did and did not (a) initiate and (b) discontinue antiplatelet therapy at index admission discharge

Total ASCVD	Initiation		Discontinuation	
	Yes	No	Yes	No
Total number (%)	126 338 (83.8)	24 390 (16.2)	28 343 (22.4)	97 995 (77.6)
Age on discharge, years (mean, SD)	66.3 (12.4)	67.5 (14.2)	67.7 (12.9)	65.8 (12.2)
SIMD quintile (2009)*				
5 (least deprived)	15.4	15.4	15.4	15.3
4	18.3	17.8	18.5	18.3
3	20.5	20.3	21.2	20.3
2	22.3	22.2	22.2	22.3
1 (most deprived)	23.6	24.3	22.7	23.8
ASCVD-related hospitalisation prior to 1 October 2009	37.8	43.4	56.0	55.3
Charlson Comorbidity Index	9.7	8.8	20.4	18.2
0 (no comorbidities)†	18.7	22.1	44.2	48.1
1	47.2	43.5	19.5	20.2
2	20.0	16.9	8.8	8.0
3	8.2	9.3	7.2	5.7
4 or more comorbidities	6.0	8.2	20.4	18.2
Mental health inpatient/day case 12 months prior to index admission	1.6	3.0	2.7	1.2
At least one antiplatelet prescription in last 12 months prior to index admission‡	53.9	38.0	31.6	33.4
ASCVD type				
MI	93.7	6.3	18.7	81.3
Stroke	83.0	17.0	24.0	76.0
PAD	68.0	32.0	27.7	72.3
Other ASCVD	80.6	19.4	23.9	76.1

* SIMD is a relative measure of deprivation across Scottish data zones.

† Absence of comorbidities as defined by CCI: in the case of the total ASCVD and other ASCVD populations, this means that individuals were not hospitalised for any of the 17 specified conditions. In the case of the MI, stroke and PAD populations, every individual has at least one CCI comorbidity, their index condition (ie, MI, stroke or PAD), thus absence of comorbidity is not applicable (N/A).

‡ For individuals with index hospitalisations in 2009, information on prior medication use is available from 1 April 2009 and onwards, thereby contributing a minimum of 6 months and up to 12 months of medication history. For all discharges recorded after 1 April 2010, medication history is available for 12 months prior to index admission.

ASCVD, atherosclerotic cardiovascular disease; CCI, Charlson Comorbidity Index; MI, myocardial infarction; PAD, peripheral arterial disease; SIMD, Scottish Index of Multiple Deprivation

**Figure 2:**
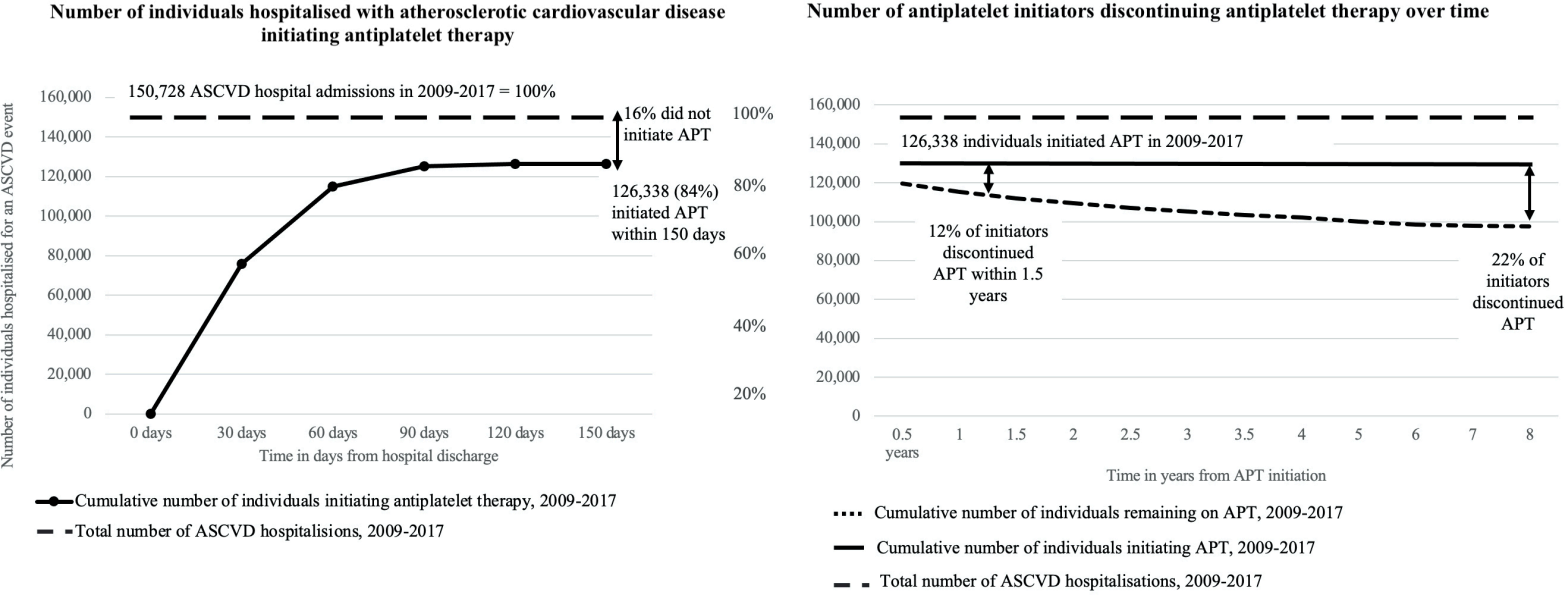
Antiplatelet initiation and discontinuation rates following atherosclerotic cardiovascular disease event. APT, antiplatelet therapy; ASCVD, atherosclerotic cardiovascular disease.

Of these individuals, 126 338 (84%) initiated APT (figure 2). The duration of the dispensed medication supply ranged from 7 to 14 days (1% of initiators), to 28 days (29%), 56 days (68%), 84 days (1.5%) and 85–180 days (0.5%); no APT was dispensed for duration >180 days. The initiation rate increased minimally from 82% in 2009–2011 to 84% in 2015–2017. Uptake varied by ASCVD type, from 68% of PAD, to 83% of ischaemic stroke and 94% of patients with MI (online supplemental figures C-F). The types of APT prescribed in 2015–2017 were as follows (table 2): among patients with MI, 74% of initiators used DAPT (MI with PCI: 82%; MI without PCI: 72%), the remainder (26%) being prescribed monotherapy. This constitutes an increase of 18% in DAPT use since 2009–2011 (p<0.001) (MI with PCI: 14%; MI without PCI: 19%). Specifically, 56% of DAPT initiators were prescribed aspirin+ticagrelor, the remainder receiving aspirin+clopidogrel (42%) and aspirin+prasugrel (2%) combinations. Monotherapy initiators were prescribed aspirin (44%), clopidogrel (33%) and ticagrelor (23%). In line with clinical guideline recommendations,^9 11^ almost all patients with stroke (98%) used monotherapy (clopidogrel: 94%; aspirin: 5%; ticagrelor: 1%). PAD initiators were prescribed aspirin (70%), followed by clopidogrel (26%) and DAPT aspirin+clopidogrel (4%). Among initiators with other ASCVD, 76% were prescribed monotherapy (other ASCVD with PCI: 50%; without PCI: 86%), and 24% DAPT (with PCI: 50%; without PCI: 14%). DAPT use increased by 7% since 2009–2011 (p<0.001) (other ASCVD with PCI: 13%; without PCI: 1%). Aspirin was the most frequently prescribed monotherapy (65%), followed by clopidogrel (33%) and ticagrelor (1%). DAPT combinations included aspirin+clopidogrel (56%), aspirin+ticagrelor (42%), other (2%).

**Table 2:** Proportion of individuals initiating antiplatelet therapy, by atherosclerotic cardiovascular disease type and antiplatelet therapy type

Proportion of individuals (in %) initiating APT in 2015–17	MI	Ischaemic stroke	PAD	Other ASCVD
DAPT	74MI with PCI: 82MI without PCI: 72	2	4	24ASCVD with PCI: 50ASCVD without PCI: 14
Breakdown by DAPT type (total=100%)				
Aspirin+ticagrelor	56	0	0	42
Aspirin+clopidogrel	42	100	100	56
Aspirin+prasugrel/other	2	0	0	2
Monotherapy	26	98	96	76ASCVD with PCI: 50ASCVD without PCI: 86
Breakdown by monotherapy type (total=100%)				
Aspirin	44	5	72	65
Clopidogrel	33	94	28	33
Ticagrelor	23	1	0	1
Prasugrel/Other	0	0	0	1

APT, antiplatelet therapy; ASCVD, atherosclerotic cardiovascular disease; DAPT, dual-antiplatelet therapy; MI, myocardial infarction; PAD, peripheral arterial disease; PCI, percutaneous coronary intervention

**Figure 3:**
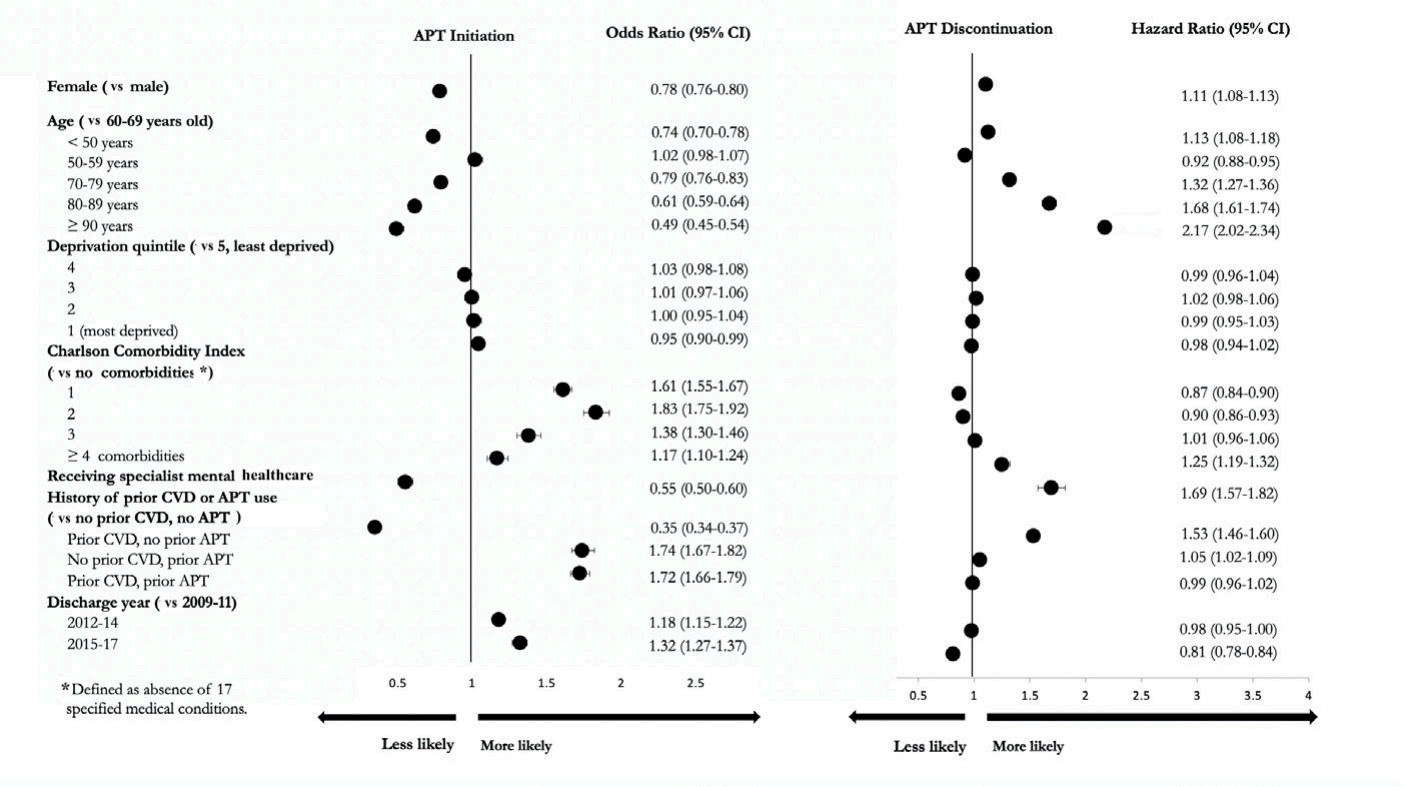
Associations of patient characteristics with antiplatelet therapy initiation and discontinuation. Please note that the CCI results presented in this figure are not easily interpretable due to index MI, stroke and PAD included among the CCI-eligible comorbidities, leaving the remaining index ASCVD conditions in the comparator ‘no comorbidity’ category. APT, antiplatelet therapy; ASCVD, atherosclerotic cardiovascular disease; CCI, Charlson Comorbidity Index; CVD, cardiovascular disease; MI, myocardial infarction; PAD, peripheral arterial disease.

Women were 22% less likely to initiate APT than men overall (OR 0.78 (95% CI 0.76 to 0.80)), with a gap of 9% for MI, 11% for ischaemic stroke and 31% for other ASCVD. No statistically significant difference by sex was observed for PAD. Compared with individuals aged 60–69 years, those below the age of 50 years and those older than 69 years were significantly less likely to initiate APT, with the likelihood of initiation decreasing as age increased beyond 70 years (<50 years: OR (95% CI) 0.74 (0.70 to 0.78); 70–79 years: 0.79 (0.76 to 0.83); 80–89 years: 0.61 (0.59 to 0.64); 90 years or older: 0.49 (0.45 to 0.54)). Patients living in the most deprived areas were 5% less likely to initiate APT compared with those in the least deprived areas (OR 0.95 (95% CI 0.90 to 0.99). Patients with previous hospitalisations at a mental health specialty were 45% less likely to initiate APT (OR 0.55 (95% CI 0.50 to 0.60)) (figure 3). In addition, a larger number of comorbidities was associated with a lower likelihood of individuals initiating APT. For example, patients with MI and stroke with two or more comorbidities had, respectively, 42% and 15% lower odds of initiating APT compared with individuals without comorbidities (MI: OR 0.58 (95% CI 0.51 to 0.65); stroke: OR 0.85 (95% CI 0.76 to 0.94)). Please see online supplemental table H for the associations between comorbidities and APT initiation by ASCVD type.

### Dual-antiplatelet therapy following percutaneous coronary intervention

Among the 19 711 individuals with ASCVD who underwent PCI, 97% initiated APT, however, only 68% of patients who underwent PCI between 2015 and 2017 received guideline-recommended DAPT, the remainder being prescribed monotherapy. Specifically, 65% of APT initiators with PCI and a drug-eluting stent (DES), 70% of APT initiators with PCI and non-DES stent and 70% of initiators with PCI without stent initiated DAPT between 2015 and 2017, the remainder using APT monotherapy. DAPT initiation rates in these three groups increased significantly compared with the rates recorded between 2009 and 2011: by 9% in PCI+DES (p<0.001), by 21% in PCI+non-DES (p<0.001) and by 24% in PCI without stent (p<0.001). Overall, the most frequently prescribed DAPT combination in the years 2015–2017 was aspirin+clopidogrel (51%), followed by aspirin+ticagrelor (47%) and aspirin+prasugrel (2%).

In addition, an analysis into the association between patient characteristics and initiation of DAPT versus antiplatelet monotherapy among individuals who underwent PCI showed that age, socioeconomic status, presence of comorbidities, history of ASCVD and APT use and calendar year played an important role in this relationship. For example, compared with patients aged 60–69 years, patients below the age of 50 years were 22% more likely to initiate DAPT (OR 1.22 (95% CI 1.07 to 1.38)), while patients aged 70–79 years were 17% less likely (OR 0.83 (95% CI 0.75 to 0.91)). Individuals living in the most deprived areas had 18% lower odds of initiating DAPT compared with those residing in the least deprived areas (OR 0.82 (95% CI 0.73 to 0.92)) (online supplemental table I).

### Antiplatelet therapy adherence, discontinuation and re-initiation

While on treatment, 92% of APT users were adherent. However, 22% of APT users discontinued treatment at some point in time and, of those who discontinued, half (51%) did so within 1.5 years since initiation, and 79% within 3.5 years (figure 2). Discontinuation rates varied by ASCVD type, ranging from 19% for patients with MI, to 24% for patients with stroke and 28% for patients with PAD. Of the individuals who discontinued therapy, only 10 517 individuals (37%) re-initiated therapy at some point in time after their initial treatment gap of 180 days. On average, individuals re-initiated APT about 1.1 years after APT discontinuation.

Patient characteristics associated with APT discontinuation were similar to those related to not initiating APT. Specifically, women had a 11% higher hazard of APT discontinuation than men (HR 1.11, 95% CI 1.08 to 1.13). A U-shaped association with age was observed: compared with individuals aged 60–69 years, those aged <50 years and those aged 70–79 years had, respectively, 13% (1.13 (95% CI 1.08 to 1.18)) and 32% (HR 1.32 (95% CI 1.27 to 1.36)) higher hazard of discontinuation, with the hazard increasing further in individuals aged 80 years or older. Patients with a history of mental health-related hospitalisation had a 69% higher hazard of discontinuing APT (HR 1.69 (95% CI 1.57 to 1.82)) (figure 3). An increase in the number of physical comorbidities was also associated with an increased hazard of APT discontinuation. For example, patients with MI with comorbidities in addition to MI had a greater hazard of discontinuing APT compared with patients with MI only, with the relative hazard increasing as the number of comorbidities increases (eg, one extra comorbidity: HR 1.11 (95% CI 1.05 to 1.17); ≥3 comorbidities: HR 1.66 (95% CI 1.54 to 2.04)) (online supplemental table J). No statistically significant association between socioeconomic status and the hazard of discontinuing APT was observed.

## Discussion

This population-wide study following individuals hospitalised for ASCVD in Scotland found suboptimal APT use at multiple treatment stages. Suboptimal APT use was more prevalent in patients discharged for non-MI ASCVD events, particularly ischaemic stroke and PAD. While only 6% of patients with MI did not initiate treatment, 17% of patients with stroke and 32% of patients with PAD did not initiate APT. Furthermore, 32% of patients who underwent PCI in 2015–2017 did not receive guideline-recommended DAPT. The majority of APT initiators were adherent while on treatment (92%), however, almost a quarter (22%) discontinued treatment. Of patients who stopped treatment, only 37% re-initiated. Patient groups at high risk of suboptimal APT treatment included women, patients aged <50 years or >70 years, patients with multiple comorbidities and those with previous mental health-related hospitalisation.

### Management of peripheral arterial disease, ischaemic stroke and other high-risk patients

Considerable variability in therapy use was observed by ASCVD type. Despite evidence from the Antithrombotic Treatment Trialists’ (ATT) Collaboration meta-analyses showing that APT reduces serious vascular events in patients with stroke and PAD, the treatment rates for stroke and PAD were markedly lower than for MI.

Previous studies in secondary prevention of ischaemic stroke showed that APT like aspirin effectively decreased risks of subsequent ischaemic stroke, while potentially increasing risks of haemorrhagic stroke, intracranial and extracranial bleeds and major gastrointestinal diseases.^1 16^ However, evidence from the ATT Collaboration meta-analyses show that the benefits of aspirin for secondary prevention heavily outweigh the potential risks of haemorrhagic stroke and the excess risk of major extracranial bleeding.^1 17^ A detailed analysis of the International Stroke Trial suggested that much of this excess bleeding occurred when APT was used in conjunction with heparin.^1 18^ Therefore, PAD-focused and stroke-focused provider training and targeted patient education may improve APT use.

### The roles of sex and age

Sex played a significant role, with women being 22% less likely to initiate and 11% more likely to discontinue APT than men. Few studies examined this relationship and made similar observations, with women being less likely to be prescribed treatment (eg, at hospital discharge^19^,^21^), while also being more likely to decline and/or discontinue treatment.^22 23^ Large meta-analyses by the ATT Collaboration did not find sex differences in the effect of aspirin for the secondary prevention of CVD, reporting a 19% relative reduction in serious vascular events for both sexes, while similarly increasing the risk of bleeding in women and men.^1 17^ Poorer adherence to ASCVD therapies among women has been, however, reported both in active and placebo treatment arms of clinical trials,^24^ indicating that broader factors play a role. Differences in CVD symptoms at presentation and diagnosis and in risk perception may have influenced physicians’ APT prescribing. Further qualitative and quantitative studies are required to investigate physician-related and patient-related factors contributing to suboptimal APT treatment in women. In the meantime, closer attention should be paid to the management of women with ASCVD.

This study also found a U-shaped relationship between age and APT use, with younger individuals below 50 years and individuals aged 70 years or older being less likely to initiate treatment, while also having a higher hazard of treatment discontinuation than those aged 60–69 years. As is the case for sex, further research is required to understand the underlying drivers of this association. Previous studies have demonstrated a positive association between older age and haemorrhagic stroke risk, which increased exponentially from the seventh decade and was associated with increased mortality.^25 26^ Similarly, suboptimal use among individuals below age 50 years may be due to a consideration of the trade-off between bleeding risks with long-term APT use and the risks of subsequent events. Therefore, it is possible that a trade-off between efficacy and safety has been considered by prescribing physicians. However, meta-analyses by the ATT Collaboration showed that APT significantly reduced risks of vascular events, irrespective of age.^27^ Therefore, further efforts are required to train both providers and patients on the safety and efficacy of APT.

### The role of other patient characteristics

The presence of comorbidities in patients with ASCVD was associated with suboptimal APT use, even though these patients are likely to be at even higher risk of future cardiovascular events. Similar findings were observed in previous nationwide cohort studies.^28^ Some comorbidities may elevate bleeding risks, which could explain the lower utilisation rates in patients with physical comorbidities. For example, a meta-analysis of randomised controlled trials investigated potential risk factors of major bleeding events and found that chronic obstructive pulmonary disease and diabetes may constitute probable risk factors.^29^ However, current risk factors assessed in validated bleeding risk assessments such as the Predicting Bleeding Complication in Patients Undergoing Stent Implantation and Subsequent Dual Antiplatelet Therapy do not include comorbidities.^30^ Furthermore, this study showed that, independently of physical comorbidities, individuals with a history of hospital admission for specialist mental care had 45% lower odds of initiating and 70% higher hazard of discontinuing APT compared with individuals without such history. Considering the large APT treatment gap in individuals with more severe mental health cases in our study, it is reasonable to propose that patients with milder mental health conditions treated in primary care, such as anxiety and moderate depression, may also be suboptimally treated with APT. Further research is required to examine the association between gradients of mental health conditions and APT use.

### Use of dual-antiplatelet therapy in patients undergoing PCI

Despite clinical guidelines recommending DAPT for the first 12 months in patients treated with PCI since 2013, only about 65%–70% of initiators treated with PCI used DAPT in 2015–2017, the remainder still being prescribed monotherapy. DAPT reduces major adverse cardiovascular events (MACE) in patients undergoing PCI with DES, although at the expense of an increased risk of major bleeding.^31^ Shorter durations of DAPT (3–6 months) were previously reported to reduce bleeding rates,^32^ while longer durations (18–48 months) yielded higher reduction in MACE but at a higher risk of major bleeding, highlighting the trade-off between efficacy and safety.^31^ While further research is required to identify which patients based on individual ischaemic and bleeding risk could benefit from shorter DAPT use, overall, the incidence of major bleeding is relatively low^17 33^ and DAPT is still recommended in patients treated with PCI,^34^ thereby highlighting significant treatment gaps in the current study population which have also been observed in other health systems.^5 35 36^ In addition, this study showed that the odds of DAPT initiation were lower for patients of older age, higher socioeconomic deprivation and with prior history of ASCVD and APT use, that is, factors that besides older age are not related to safety of DAPT use.

### Long-term associations between suboptimal APT use and health outcomes

Meta-analyses of randomised controlled trials showed that aspirin yields relative risk reductions for future ASCVD events of 30% for MI, 20% for ischaemic stroke, 30% for other vascular events and 18% for all-cause mortality among individuals with established ASCVD treated with prolonged aspirin therapy.^1^ With 16% of patients with ASCVD not initiating treatment, in addition to 22% discontinuing treatment over time, about 20% of patient-years were not covered by APT after an event. About 25% of events in those patient years (ie, 5% of all major ASCVD events in the entire ASCVD population) might have been prevented if APT had been used. The impact of poor adherence is likely to be even greater if suboptimal therapy in the same patients extends to other proven secondary prevention treatments such as statins, beta-blockers and renin-angiotensin system blockers. As previously shown, the use of statin therapy for the secondary prevention of ASCVD in Scotland is suboptimal and an additional 6%–10% of all subsequent ASCVD events might have been prevented if, respectively, moderate-intensity or high-intensity statins had been used.^8^ Therefore, approximately 10%–15% of all ASCVD events might have been prevented if both APT and statin therapy had been used.

### Strengths and limitations

The dataset has major strengths in that it captures the entire national population of Scotland, including every individual ever hospitalised for an ASCVD event and is, therefore, highly representative. It allowed for a detailed analysis into the extent of APT use at different pathway stages. Several limitations should be acknowledged. The PIS dataset used for this study only included information for individuals who were dispensed treatment and, therefore, we could not differentiate between treatment not being prescribed and treatment being prescribed but not dispensed. Additionally, PIS does not contain information on the medication indication. Furthermore, we had data only on medication being collected but not whether patients actually used it, although patients who continue to regularly collect but not use treatment are likely rare. The data only include prescriptions issued in primary care. However, as APT issued during hospitalisation would typically only last a few weeks, requiring patients to continue treatment in primary care, this is unlikely to represent a major deficiency. Due to the limitations of the data and the need to define initiation of APT, it was not possible to assess if individuals who died within 150 days of index discharge were more, equally or less likely to initiate APT than those who survived this time period. Patient characteristics were derived from hospital records only, which do not capture patient’s full medical history and diagnoses. Hence, the actual proportion of individuals with physical and mental comorbidities will be underestimated. Similarly, it was not possible to fully account for individuals who were not prescribed or who were asked to discontinue treatment on clinically acceptable grounds, such as terminal illness, non-serious bleeding or any contraindications to APT. To partly mitigate this limitation, the analyses excluded all individuals who died within 150 days of hospital discharge. Besides patients hospitalised for atrial fibrillation, other patient groups likely requiring long-term oral anticoagulation (eg, patients with cancer with venous thromboembolism, and those with mechanical valve prostheses) were not excluded as study data only included records for APT dispenses, however, the total number of these patients is expected to be small. The relevance of further patient characteristics that may be relevant to the likelihood of using APT, such as patients’ ethnicity and marital status, could not be studied due to large numbers of missing observations. The data also do not provide insights into clinicians’ characteristics and management of a patient, clinicians’ and patients’ beliefs, preferences or risk perceptions of APT. Lastly, the clinical guideline recommendations and thresholds for APT use may differ across countries, which needs to be considered in assessments of care quality and the implementation of advanced patient technologies to support patient care.

## Conclusions

Although the majority of individuals hospitalised for an ASCVD are adherent to APT while on treatment, the proportions of individuals who initiate APT following an ASCVD event, in addition to persisting in its use, remain suboptimal. While treatment of MI is good, with high levels of DAPT, the use of APT following ischaemic stroke and PAD is poor. Women, individuals aged 50 years or younger and those aged 70 years and older, those with multiple comorbidities and previous mental health-related hospital admission and those living in the most deprived areas are at increased risk of suboptimal APT use for the secondary prevention of ASCVD. Suboptimal antiplatelet use leads to higher risks of recurrent ASCVD events and mortality, which needs to be addressed by clinicians and policy-makers.

## supplementary material

10.1136/bmjqs-2023-016520 online supplemental file 1

## Data Availability

Data may be obtained from a third party and are not publicly available.

## References

[1] Antithrombotic Trialists’ Collaboration (2002). Collaborative meta-analysis of randomised trials of antiplatelet therapy for prevention of death, myocardial infarction, and stroke in high risk patients. BMJ.

[2] Chiarito M, Sanz-Sánchez J, Cannata F (2020). Monotherapy with a P2Y12 inhibitor or aspirin for secondary prevention in patients with established atherosclerosis: a systematic review and meta-analysis. The Lancet.

[3] Wallentin L, Becker RC, Budaj A (2009). Ticagrelor versus clopidogrel in patients with acute coronary syndromes. N Engl J Med.

[4] Verro P, Gorelick PB, Nguyen D (2008). Aspirin plus dipyridamole versus aspirin for prevention of vascular events after stroke or TIA. Stroke.

[5] Green A, Pottegård A, Broe A (2016). Initiation and persistence with dual antiplatelet therapy after acute myocardial infarction: a Danish nationwide population-based cohort study. BMJ Open.

[6] Hoer A, Behrendt S, Schmidt T (2013). Healthcare utilization of patients with acute coronary syndrome in Germany. Cardiol Res.

[7] Glader E-L, Sjölander M, Eriksson M (2010). Persistent use of secondary preventive drugs declines rapidly during the first 2 years after stroke. Stroke.

[8] Thalmann I, Preiss D, Schlackow I (2023). Population-wide cohort study of Statin use for the secondary cardiovascular disease prevention in Scotland in 2009–2017. Heart.

[9] Scottish Intercollegiate Guidelines Network (2008). Risk estimation and the prevention of cardiovascular disease A national clinical guideline.

[10] Scottish Intercollegiate Guidelines Network (2016). SIGN 148 acute coronary syndrome. Healthcare improvement Scotland. https://www.sign.ac.uk/assets/sign148.pdf.

[11] Scottish Intercollegiate Guidelines Network (SIGN) (2017). SIGN 149 - risk estimation and the prevention of cardiovascular disease, A national clinical guideline. https://www.sign.ac.uk/assets/sign149.pdf.

[12] Malo S, Aguilar-Palacio I, Feja C (2017). Different approaches to the assessment of adherence and persistence with cardiovascular-disease preventive medications. Curr Med Res Opin.

[13] Clancy ZA (2013). MPR and PCD: implications for interpretation of adherence research results. Value in Health.

[14] Cox DR (1972). Regression models and life-tables. Journal of the Royal Statistical Society: Series B (Methodological).

[15] Schoenfeld D (1982). Partial residuals for the proportional hazards regression model. Biometrika.

[16] García Rodríguez LA, Martín-Pérez M, Hennekens CH (2016). Bleeding risk with long-term low-dose aspirin: a systematic review of observational studies. PLoS One.

[17] Antithrombotic Trialists’ Collaboration (2009). Aspirin in the primary and secondary prevention of vascular disease: collaborative meta-analysis of individual participant data from randomised trials. Lancet.

[18] International Stroke Trial Collaborative Group (1997). The International stroke trial (IST): a randomised trial of aspirin, subcutaneous heparin, both, or neither among 19 435 patients with acute ischaemic stroke. The Lancet.

[19] Lawesson SS, Alfredsson J, Fredrikson M (2012). Time trends in STEMI—improved treatment and outcome but still a gender gap: a prospective observational cohort study from the SWEDEHEART register. BMJ Open.

[20] Redfors B, Angerås O, Råmunddal T (2015). Trends in gender differences in cardiac care and outcome after acute myocardial infarction in Western Sweden: a report from the Swedish web system for enhancement of evidence-based care in heart disease evaluated according to recommended therapies (SWEDEHEART). J Am Heart Assoc.

[21] Radovanovic D, Nallamothu BK, Seifert B (2012). Temporal trends in treatment of ST-elevation myocardial infarction among men and women in Switzerland between 1997 and 2011. Eur Heart J Acute Cardiovasc Care.

[22] Sáez ME, González-Pérez A, Johansson S (2015). Patterns of antiplatelet therapy in patients who have experienced an acute coronary event: a descriptive study in UK primary care. J Cardiovasc Pharmacol Ther.

[23] Liu X, He X, Wu J (2019). Initiation and persistence with antiplatelet agents among the patients with acute coronary syndromes: a retrospective, observational database study in China. Patient Prefer Adherence.

[24] Lau ES, Braunwald E, Morrow DA (2021). Sex, permanent drug discontinuation, and study retention in clinical trials. Circulation.

[25] Roe MT, Goodman SG, Ohman EM (2013). Elderly patients with acute coronary syndromes managed without revascularization: insights into the safety of long-term dual antiplatelet therapy with reduced-dose Prasugrel versus standard-dose clopidogrel. Circulation.

[26] Mehran R, Pocock S, Nikolsky E (2011). Impact of bleeding on mortality after percutaneous coronary intervention results from a patient-level pooled analysis of the REPLACE-2 (randomized evaluation of PCI linking Angiomax to reduced clinical events), ACUITY (acute Catheterization and urgent intervention triage strategy), and HORIZONS-AMI (harmonizing outcomes with Revascularization and Stents in acute myocardial infarction) trials. JACC Cardiovasc Interv.

[27] Antiplatelet Trialists’ Collaboration (1994). Collaborative overview of randomised trials of antiplatelet therapy prevention of death, myocardial infarction, and stroke by prolonged antiplatelet therapy in various categories of patients. BMJ.

[28] Prami T, Khanfir H, Deleskog A (2016). Clinical factors associated with initiation of and persistence with ADP receptor-inhibiting oral antiplatelet treatment after acute coronary syndrome: a nationwide cohort study from Finland. BMJ Open.

[29] Nguyen KA, Eadon MT, Yoo R (2021). Risk factors for bleeding and clinical ineffectiveness associated with clopidogrel therapy: a comprehensive meta-analysis. Clin Transl Sci.

[30] Costa F, van Klaveren D, James S (2017). Derivation and validation of the predicting bleeding complications in patients undergoing Stent implantation and subsequent dual antiplatelet therapy (PRECISE-DAPT) score: a pooled analysis of individual-patient datasets from clinical trials. The Lancet.

[31] Degrauwe S, Pilgrim T, Aminian A (2017). Dual antiplatelet therapy for secondary prevention of coronary artery disease. Open Heart.

[32] Giacoppo D, Matsuda Y, Fovino LN (2021). Short dual antiplatelet therapy followed by P2Y12 inhibitor monotherapy vs. prolonged dual antiplatelet therapy after percutaneous coronary intervention with second-generation drug-eluting stents: a systematic review and meta-analysis of randomized clinical trials. Eur Heart J.

[33] Ahmed B, Dauerman HL (2013). Bleeding, and coronary intervention. Circulation.

[34] National Institute for Health Care and Excellence (NICE) (2020). Acute coronary syndromes, NICE guideline [Ng185]. https://www.nice.org.uk/guidance/ng185.

[35] Sheikh Rezaei S, Geroldinger A, Heinze G (2017). Clopidogrel, prasugrel, or ticagrelor use and clinical outcome in patients with acute coronary syndrome: a nationwide long-term registry analysis from 2009 to 2014. Int J Cardiol.

[36] Sahlén A, Varenhorst C, Lagerqvist B (2016). Outcomes in patients treated with ticagrelor or clopidogrel after acute myocardial infarction: experiences from SWEDEHEART registry. Eur Heart J.

