# Supplementary material for: Quality of care for secondary cardiovascular disease prevention in 2009–2017: population-wide cohort study of antiplatelet therapy use in Scotland
Source: BMJ Qual Saf. 2023 Sep 29;33(11):e016520. doi: 10.1136/bmjqs-2023-016520 (PMC7616486; doi:10.1136/bmjqs-2023-016520)
Supplement: online supplemental file 1 [file bmjqs-33-11-s001.pdf]

SUPPLEMENTAL MATERIAL

Quality of care for secondary cardiovascular disease prevention in 2009-2017: population-wide cohort study of antiplatelet therapy use in Scotland

TABLES

Table A Overview of the four linked NHS Scotland datasets used in this study

**Dataset**	**Examples of information included**
General/Acute Inpatient and Day Case (SMR01)	Contains episode-level data on inpatient and day case discharges and covers all individuals (residents and non-residents of Scotland) who receive care in non-obstetric, non-psychiatric NHS general acute specialties and hospitals.<br><br>Each episode contains information on diagnoses using International Classification of Diseases (ICD) codes, operations and procedures, as well as patient and hospital details, such as patient demographics, episode management, socio-economic and geographical information.
Mental Health Inpatient and Day Case (SMR04)	Contains episode-level data on individuals admitted to care in NHS hospital mental health specialties and psychiatric hospitals.<br><br>Each episode contains information on diagnoses using International Classification of Diseases (ICD) codes, as well as patient and hospital details, such as patient demographics, episode management.
National Records of Scotland (NRS deaths)	Contains death-related information (date of death, age and specific causes of death classified under ICD-10)
Prescribing Information System (PIS)	Contains general information on all medicines that were both prescribed and dispensed in the community (i.e., primary care) in Scotland (e.g., information on prescribed items such as formulation code, strength, dose instructions, quantity prescribed; details of the prescribing individuals, practice and dispenser: list size of practice, geographical location). It does not contain information on medicines that were prescribed but not dispensed.

Table B Overview of the definitions of atherosclerotic cardiovascular disease types by International Classification of Diseases Revision 10 code

**ASCVD Type**	**ICD-10 codes**
Total ASCVD	I20-25, I63- 67, I70-71, and I73.9 recorded in the primary diagnostic field
Myocardial infarction	I21-I22 and min. 1 day hospital length of stay recorded in the primary diagnostic field
Ischaemic stroke	I63-I64 and min. 1 day hospital length of stay recorded in the primary diagnostic field
Peripheral arterial disease	I71, I73.9 recorded in the primary diagnostic field
Other ASCVD	I20, I23, I24, I25.0-1, I25.8-9, I65-I67, I70 recorded in the primary diagnostic field
Atrial fibrillation	I48X recorded in the primary and secondary diagnostic fields

ASCVD: atherosclerotic cardiovascular disease; ICD-10: International Classification of Diseases Revision 10.

Table C Overview of Anatomical Therapeutic Chemical and British National Formulary codes for antiplatelet therapies

**Antiplatelet**	**ATC code**	**BNF Code**
Aspirin	N02BA01	0209000A0
Clopidogrel	B01AC04	0209000C0
Dipyridamole	B01AC07	0209000L0
Dipyridamole & Aspirin	B01AC30	0209000V0
Prasugrel	B01AC22	0209000Y0
Ticagrelor	B01AC24	0209000Z0

ATC: Anatomical Therapeutic Chemical; BNF: British National Formulary

Table D Detailed overview of the list of conditions and associated International Classification of Diseases Revision 10 codes included in the Charlson Comorbidity Index

Condition included in the Charlson Comorbidity Index	ICD-10 code
1. AIDS/HIV	B20.X - B22.X, B24.X
2. Any malignancy, including lymphoma and leukaemia, except malignant neoplasm of skin	C00.X - C26.X, C30.X - C34.X, C37.X - C41.X, C43. X, C45. X - C58.X, C60. X - C76.X, C81.X - C85.X, C88.X, C90.X - C97.X
3. Cerebrovascular disease	G45.X, G46.X, H34.0, I60.X - I69.X
4. Chronic pulmonary disease	I27.8, I27.9, J40.X - J47.X, J60.X - J67.X, J68.4, J70.1, J70.3
5. Congestive heart failure	I09.9, I11.0, I13.0, I13.2, I25.5, I42.0, I42.5 - I42.9, I43.X, I50.X, P29.0
6. Dementia	F00.X - F03.X, F05.1, G30.X, G31.1
7. Diabetes without chronic complication	E10.0, E10.1, E10.6, E10.8, E10.9, E11.0, E11.1, E11.6, E11.8, E11.9, E12.0, E12.1, E12.6, E12.8, E12.9, E13.0, E13.1, E13.6, E13.8, E13.9, E14.0, E14.1, E14.6, E14.8, E14.9
8. Diabetes with chronic complication	E10.2 - E10.5, E10.7, E11.2 - E11.5, E11.7, E12.2 - E12.5, E12.7, E13.2 - E13.5, E13.7, E14.2 - E14.5, E14.7
9. Hemiplegia or paraplegia	G04.1, G11.4, G80.1, G80.2, G81.X, G82.X, G83.0 - G83.4, G83.9
10. Metastatic solid tumour	C77.X - C80.X
11. Mild liver disease	B18.X, K70.0 - K70.3, K70.9, K71.3 - K71.5, K71.7, K73.X, K74.X, K76.0, K76.2 - K76.4, K76.8, K76.9, Z94.4
12. Moderate or severe liver disease	I85.0, I85.9, I86.4, I98.2, K70.4, K71.1, K72.1, K72.9, K76.5, K76.6, K76.7
13. Myocardial infarction	I21.X, I22.X, I25.2
14. Peptic ulcer disease	K25.X - K28.X
15. Peripheral vascular disease	I70.X x, I71.X, I73.1, I73.8, I73.9, I77.1, I79.0, I79.2, K55.1, K55.8, K55.9, Z95.8, Z95.9
16. Renal disease	I12.0, I13.1, N03.2 - N03.7, N05.2 - N05.7, N18.X, N19.X, N25.0, Z49.0 - Z49.2, Z94.0, Z99.2
17. Rheumatic disease	M05.X, M06.X, M31.5, M32.X - M34.X, M35.1, M35.3, M36.0

ICD-10: International Classification of Diseases Revision 10.

Table E Baseline characteristics of individuals hospitalised for an atherosclerotic cardiovascular disease-related event at index discharge

	**Total ASCVD**
**N**	150,728
**Age on discharge, years** (mean, SD)	66.5 (12.7)
**Female**	58,321 (38.7)
**Ethnic group**	
White	128,323 (85.0)
Other	2,250 (1.5)
Missing	20,246 (13.5)
**SIMD deprivation quintile (2009)** [a]	
5 (least deprived)	23,149 (15.4)
4	27,507 (18.3)
3	30,858 (20.4)
2	33,527 (22.2)
1 (most deprived)	35,687 (23.7)
**ASCVD-related hospitalisation prior to 1 October 2009**	58,238 (39.0)
**Charlson Comorbidity Index (within 12 months prior and incl. index event)**	
0 (no-comorbidities) [b]	28,962 (19.2)
1	70,225 (46.6)
2	29,389 (19.5)
3	12,558 (8.3)
4 or more comorbidities	9,594 (6.4)
**Mental health inpatient/day case within 12 months prior to index admission**	2,692 (1.8)
**At least one antiplatelet prescription in last 12 months prior to index admission** [c]	77,392 (51.3)
**ASCVD Type**	
MI	45,841 (30.4)
Stroke	26,867 (17.8)
PAD	14,307 (9.5)
Other ASCVD	63,713 (42.3)
**Deaths recorded in the time period between the index discharge date and the study end date on 31 December 2017**	
Total (%)	34,257 (23)
**Time to death in days (months) since index discharge (% of all deaths)**	
0-150 days (0-4.9 months)	0
151-365 days (5-12 months)	6,101 (18)
366-730 days (13-24 months)	7,625 (22)
731-1095 days (25-36 months)	6,247 (18)

	Total ASCVD
1096-1461 days (37-48 months)	5,036 (15)
1462-1826 days (49-60 months)	3,838 (11)
1827-2191 days (61-72 months)	2,813 (8)
2192-2556 days (73-84 months)	1,746 (5)
2557-2992 days (85-96 months)	851 (2)

ASCVD: atherosclerotic cardiovascular disease; APT: antiplatelet therapy; CCI: Charlson Comorbidity Index; MI: myocardial infarction; PAD: peripheral arterial disease; SD: standard deviation; SIMD: Scottish Index of Multiple Deprivation.

[a] SIMD is a relative measure of deprivation across Scottish data zones.
[b] Absence of comorbidities as defined by CCI: in the case of the total ASCVD and other ASCVD populations, this means that individuals were not hospitalised for any of the 17 specified conditions. In the case of the MI, stroke and PAD populations, every individual has at least one CCI comorbidity, their index condition (i.e., MI, stroke or PAD), thus absence of comorbidity is not applicable (N/A).
[c] For individuals with index hospitalisations in 2009, information on prior medication use is available from 1 April 2009 and onward, thereby contributing a minimum of 6 months and up to 12 months of medication history. For all discharges recorded after 1 April 2010, medication history is available for 12 months prior to index admission.

Note: The following characteristics were assessed: sex (male, female), age group at index event date (≤49, 50-59, 60-69, 70-79, 80-89, ≥90 years), deprivation quintiles based on the SIMD (where 1 indicates most deprived and 5, least deprived) (11), CCI (defined as the number of comorbidities reported in the hospital admissions data within the 12 months prior to and including index admission discharge, where ‘no comorbidities’ is defined as the absence of 17 specified medical conditions (see **Supplements, Table D**): no comorbidities, 1, 2, 3, or ≥ 4 comorbidities), inpatient or outpatient admission to a mental health specialty or psychiatric hospital within 12 months prior to index ASCVD admission (yes, no), history of any previous ASCVD event and/or previous antiplatelet therapy use in the last 12 months prior to index ASCVD admission (no history of ASCVD and no prior use of APT; history of ASCVD and no prior use of APT; no history of ASCVD and prior use of APT; and history of ASCVD and prior use of APT), and discharge calendar year (2009-2011, 2012-2014, 2015-2017).

Table F Participant characteristics of antiplatelet therapy initiators and non-initiators at index admission discharge, total atherosclerotic cardiovascular disease and by atherosclerotic cardiovascular disease type

	**Total ASCVD**		**MI**		**Ischaemic stroke**		**PAD**		**Other ASCVD**	
APT initiation, results presented in %	Yes	No	Yes	No	Yes	No	Yes	No	Yes	No
**Total number (%)**	126,338 (83.8)	24,390 (16.2)	42,943 (93.7)	2,898 (6.3)	22,303 (83.0)	4,564 (17.0)	9,729 (68.0)	4,578 (32.0)	51,363 (80.6)	12,350 (19.4)
**Age on discharge, years** (mean, SD)	66.3 (12.4)	67.5 (14.2)	64.6 (13.3)	70.4 (14.8)	68.9 (13.4)	71.8 (16.1)	68.6 (10.9)	67.6 (13.4)	66.1 (11.1)	65.5 (13.7)
**Hospital length of stay, days** (mean, SD)	6.3 (11.4)	9.1 (16.0)	6.0 (6.9)	10.3 (12.1)	14.3 (18.9)	21.4 (23.1)	8.1 (13.1)	9.2 (13.4)	2..7 (7.1)	3.8 (10.0)
**Female**	37.4	45.3	34.2	42.5	46.6	51.8	35.0	35.7	36.6	47.1
**Ethnic group**										
White	85.0	85.3	85.3	85.7	84.7	85.7	86.7	86.2	84.6	84.8
Other	1.5	1.3	1.7	2.1	1.0	0.9	0.4	0.5	1.8	1.6
Missing	13.5	13.3	13.0	12.3	14.3	13.4	12.9	13.3	13.6	13.6
**SIMD quintile (2009)**[1]										
5 (least deprived)	15.4	15.4	15.2	13.8	14.9	15.6	12.9	14.1	16.1	16.1
4	18.3	17.8	18.5	15.7	17.6	16.4	18.2	19.2	18.6	18.3
3	20.5	20.3	20.5	20.3	19.8	19.7	21.1	20.7	20.7	20.3
2	22.3	22.2	22.1	22.1	22.5	22.7	24.5	22.9	21.8	21.8
1 (most deprived)	23.6	24.3	23.7	28.1	25.2	25.6	23.3	23.1	22.7	23.4
**Urban-Rural Classification**[2]										
Large urban areas	31.5	34.9	32.0	39.9	32.7	37.1	27.3	29.6	31.4	34.9
Other urban areas	9.7	8.8	9.9	8.3	9.7	8.1	9.6	9.4	9.5	8.9
Accessible small towns	4.1	3.9	4.0	3.8	3.9	4.2	4.9	3.7	4.1	3.9
Remote small towns	11.6	10.9	11.7	9.0	11.1	9.9	12.2	12.7	11.6	11.0
Accessible rural	6.7	6.3	6.4	4.9	6.1	5.6	7.6	6.4	7.0	6.8
Remote rural										

[1] SIMD is a relative measure of deprivation across Scottish data zones.

[2] Scottish Government Urban Rural Classification: (1) Large urban areas: settlements of 125,0000 or more people; (2) Other urban areas: settlements of 10,000 to 124,999 people; (3) Accessible small towns: settlements of 3,000 to 9,999 people and within 30 minutes' drive of a settlement of 10,000 or more; (4) Remote small towns: settlements of 3,000 to 9,999 people and a drive time of over 30 minutes to a settlement of 10,000 or more; (5) Accessible rural: Areas with a population of less than 3,000 people, and within a 30 minute drive time from a settlement of 10,000 or more; (6) Remote rural: Areas with a population of less than 3,000 people, and with a drive time of over 30 minutes to a settlement of 10,000 or more.

APT initiation, results presented in %	Total ASCVD		MI		Ischaemic stroke		PAD		Other ASCVD	
	Yes	No	Yes	No	Yes	No	Yes	No	Yes	No
**Charlson Comorbidity Index**	9.7	8.8	9.9	8.3	9.7	8.1	9.6	9.4	9.5	8.9
0 (no-comorbidities)[3]	18.7	22.1	N/A	N/A	N/A	N/A	N/A	N/A	45.9	43.6
1	47.2	43.5	55.2	39.7	58.7	54.6	64.7	62.7	32.2	33.2
2	20.0	16.9	27.5	27.6	22.7	22.6	17.6	16.6	13.0	12.4
3	8.2	9.3	9.9	16.5	10.9	12.7	9.7	10.2	5.2	5.9
4 or more comorbidities	6.0	8.2	7.4	16.2	7.8	10.1	8.0	10.4	3.7	4.8
**Mental health inpatient/day case 12 months prior to index admission**	1.6	3.0	1.3	4.0	2.2	4.6	1.1	1.8	1.5	2.7
**ASCVD history and APT prescription 12 months prior to index admission**[4]										
No ASCVD hospitalisation + no APT use	39.6	41.0	61.5	43.3	54.2	48.1	15.9	40.3	19.5	38.2
ASCVD hospitalisation + no APT use	6.5	21.0	8.0	16.1	8.5	16.8	5.6	26.3	4.4	21.7
No ASCVD hospitalisation + APT use	22.6	15.6	10.3	11.9	16.2	14.6	32.7	13.6	33.9	17.6
ASCVD hospitalisation + APT use	31.3	22.4	20.2	28.6	21.2	20.5	45.9	19.8	42.2	22.5
**Time to first primary care level prescription since index discharge, days** (mean, SD)[5]	18.2 (17.8)	Not applicable	14.1 (13.7)	Not applicable	16.0 (16.5)	Not applicable	25.9 (22.0)	Not applicable	21.2 (19.5)	Not applicable
**Time from prescription to dispense, days** (mean, SD)	11.5 (13.4)		11.1 (13.0)		12.7 (14.8)		11.5 (13.4)		11.2 (13.0)	

APT: antiplatelet therapy; ASCVD: atherosclerotic cardiovascular disease; CCI: Charlson Comorbidity Index; MI: myocardial infarction; PAD: peripheral arterial disease; SD: standard deviation.

[3] Absence of comorbidities as defined by CCI: in the case of the total ASCVD and other ASCVD populations, this means that individuals were not hospitalised for any of the 17 specified conditions. In the case of the MI, stroke and PAD populations, every individual has at least one CCI comorbidity, their index condition (i.e., MI, stroke or PAD), thus absence of comorbidity is not applicable (N/A).

[4] For individuals with index hospitalisations in 2009, information on prior medication use is available from 1 April 2009 and onward, thereby contributing a minimum of 6 months and up to 12 months of medication history. For all discharges recorded after 1 April 2010, medication history is available for 12 months prior to index admission.

[5] Please note that this statistic only includes individuals who initiated the prescribed treatment, as the Prescribing Information System (PIS) data do not contain prescribing information for patients who were not dispensed treatment.

Table G Characteristics of patients who did and did not discontinue antiplatelet therapy, total atherosclerotic cardiovascular disease and by atherosclerotic cardiovascular disease type

	**Total ASCVD**		**MI**		**Ischaemic stroke**		**PAD**		**Other ASCVD**	
APT discontinuation, results presented in %	Yes	No	Yes	No	Yes	No	Yes	No	Yes	No
**Total number (%)**	28,343 (22.4)	97,995 (77.6)	8,039 (18.7)	34,904 (81.3)	5,355 (24.0)	16,948 (76.0)	2,690 (27.7)	7,039 (72.3)	12,259 (23.9)	39,104 (76.1)
**Time to discontinuation** (mean, SD)	2.0 (1.7)	Not applicable	2.1 (1.7)	Not applicable	1.8 (1.7)	Not applicable	1.9 (1.8)	Not applicable	2.0 (1.8)	Not applicable
**Age on discharge**, in years (mean, SD)	67.7 (12.9)	65.8 (12.2)	67.2 (14.2)	64.0 (13.0)	70.1 (13.8)	68.6 (13.2)	69.2 (11.7)	68.4 (10.5)	66.7 (11.8)	65.8 (10.9)
**Female**	40.9	36.4	38.6	33.1	48.0	46.2	35.7	34.8	40.4	35.4
**Ethnic group**										
White	86.4	84.6	87.0	84.9	86.1	84.2	87.7	86.3	85.8	84.2
Other	1.7	1.5	1.9	1.6	1.1	0.9	0.3	0.4	2.1	1.8
Missing	11.9	13.9	11.1	13.4	12.8	14.8	12.0	13.3	12.2	14.0
**SIMD quintile (2009)**[6]										
5 (least deprived)	15.4	15.3	14.8	15.3	15.8	14.7	12.8	12.9	16.2	16.1
4	18.5	18.3	18.3	18.5	18.0	17.4	17.5	18.5	19.0	18.5
3	21.2	20.3	20.9	20.4	19.9	19.8	22.6	20.5	21.7	20.4
2	22.2	22.3	22.4	22.1	22.7	22.4	23.2	25.0	21.5	21.9
1 (most deprived)	22.7	23.8	23.6	23.8	23.6	25.7	23.9	23.1	21.6	23.1
**Urban-Rural Classification**[7]										
Large urban areas	32.5	31.3	34.1	31.6	33.8	32.4	26.1	27.7	32.2	31.1
Other urban areas	34.5	37.1	33.8	36.7	33.8	37.4	39.8	38.1	34.0	37.2
Accessible small towns	9.3	9.8	9.3	10.0	9.2	9.6	9.1	9.8	9.2	9.6
Remote small towns	4.5	3.9	4.5	3.8	4.2	3.9	5.3	4.7	4.5	4.0
Accessible rural	11.6	11.5	11.1	11.8	11.7	10.9	11.2	12.5	11.9	11.5

[6] SIMD is a relative measure of deprivation across Scottish data zones.

[7] Scottish Government Urban Rural Classification: (1) Large urban areas: settlements of 125,0000 or more people; (2) Other urban areas: settlements of 10,000 to 124,999 people; (3) Accessible small towns: settlements of 3,000 to 9,999 people and within 30 minutes' drive of a settlement of 10,000 or more; (4) Remote small towns: settlements of 3,000 to 9,999 people and a drive time of over 30 minutes to a settlement of 10,000 or more; (5) Accessible rural: Areas with a population of less than 3,000 people, and within a 30 minute drive time from a settlement of 10,000 or more; (6) Remote rural: Areas with a population of less than 3,000 people, and with a drive time of over 30 minutes to a settlement of 10,000 or more.

APT discontinuation, results presented in %	Total ASCVD		MI		Ischaemic stroke		PAD		Other ASCVD	
	Yes	No	Yes	No	Yes	No	Yes	No	Yes	No
Remote rural	7.6	6.4	7.2	6.2	6.4	6.0	8.5	7.3	8.2	6.6
**Charlson Comorbidity Index**										
0 (no-comorbidities) [8]	20.4	18.2	N/A	N/A	N/A	N/A	N/A	N/A	47.1	45.5
1	44.2	48.1	48.2	56.8	58.3	58.8	64.5	64.7	30.9	32.5
2	19.5	20.2	28.8	27.2	21.9	22.9	17.0	17.9	12.8	13.1
3	8.8	8.0	12.5	9.3	11.2	10.8	9.9	9.7	5.1	5.2
4 or more comorbidities	7.2	5.7	10.6	6.7	8.6	7.5	8.6	7.7	4.0	3.6
**Mental health inpatient/day case 12 months prior to index admission**	2.7	1.2	2.6	1.1	3.5	1.8	1.8	0.9	2.6	1.2
**ASCVD history and APT prescription in the 12 months prior to index admission[9]**										
No ASCVD hospitalisation + no APT	35.3	37.4	52.3	57.0	49.7	49.9	18.4	14.1	21.6	18.5
No ASCVD hospitalisation + APT use	8.7	7.3	11.8	16.6	17.0	21.6	7.1	6.9	6.5	6.3
ASCVD hospitalisation + no APT use	22.9	26.1	11.7	7.8	9.8	9.0	31.8	33.8	30.8	35.2
ASCVD hospitalisation + APT use	33.1	29.2	24.2	18.6	23.5	19.5	42.7	45.2	41.1	40.0

APT: antiplatelet therapy; ASCVD: atherosclerotic cardiovascular disease; Charlson Comorbidity Index; MI: myocardial infarction; PAD: peripheral arterial disease; SD: standard deviation.

[8] Absence of comorbidities as defined by CCI: in the case of the total ASCVD and other ASCVD populations, this means that individuals were not hospitalised for any of the 17 specified conditions. In the case of the MI, stroke and PAD populations, every individual has at least one CCI comorbidity, their index condition (i.e. MI, stroke or PAD), thus absence of comorbidity is not applicable (N/A).

[9] For individuals with index hospitalisations in 2009, information on prior medication use is available from 1 April 2009 and onward, thereby contributing a minimum of 6 months and up to 12 months of medication history. For all discharges recorded after 1 April 2010, medication history is available for 12 months prior to index admission.

Table H Associations of patient characteristics with antiplatelet therapy initiation among individuals with atherosclerotic cardiovascular disease, by atherosclerotic cardiovascular disease type (multivariable logistic regression models)

	**MI**		**Ischaemic stroke**		**PAD**		**Other ASCVD**	
	OR	95% CI	OR	95% CI	OR	95% CI	OR	95% CI
**Female (vs. male)**	0.91*	0.84-0.99	0.89**	0.83-0.95	1.02	0.93-1.11	0.69***	0.66-0.72
**Age (vs. 60-69 years old)**								
<50	0.83*	0.71-0.96	0.59***	0.52-0.67	0.62***	0.53-0.73	0.62***	0.57-0.66
50-59 years	1.06	0.93-1.21	0.97	0.86-1.10	0.88	0.77-1.00	0.93*	0.87-0.99
70-79 years	0.68***	0.60-0.76	0.73***	0.66-0.81	0.89*	0.80-0.99	0.87***	0.82-0.92
80-80 years	0.44***	0.38-0.49	0.54***	0.49-0.60	0.73***	0.64-0.82	0.64***	0.60-0.69
≥ 90 years	0.26***	0.22-0.32	0.43***	0.36-0.50	0.63**	0.46-0.86	0.52***	0.44-0.61
**Deprivation quintile (vs. 5, least deprived)**								
4	1.07	0.93-1.24	1.13*	1.00-1.26	1.00	0.87-1.15	1.04	0.96-1.11
3	0.93	0.81-1.06	1.06	0.95-1.18	1.09	0.95-1.25	1.06	0.98-1.13
2	0.93	0.82-1.07	1.04	0.93-1.15	1.10	0.96-1.26	1.04	0.97-1.12
1 (most deprived)	0.74***	0.65-0.85	0.99	0.89-1.10	1.03	0.90-1.18	1.04	0.97-1.11
**Charlson Comorbidity Index (vs. no comorbidities)**[1]								
1	N/A		N/A		N/A		1.04	0.99-1.20
2	0.83***	0.75-0.91	0.95	0.88-1.04	0.96	0.86-1.06	1.08*	1.00-1.15
3	0.58***	0.51-0.65	0.85**	0.76-0.94	0.85*	0.75-0.97	0.86**	0.78-0.95
4 or more comorbidities	0.49***	0.44-0.56	0.79***	0.70-0.88	0.62***	0.55-0.72	0.74***	0.67-0.83
**Receiving specialist mental health care**	0.38***	0.30-0.47	0.50***	0.42-0.60	0.70*	0.50-0.97	0.64***	0.55-0.73
**History of prior ASCVD or APT use (vs. no prior ASCVD, no prior APT)**								
Prior ASCVD, no prior APT	0.49***	0.43-0.55	0.50***	0.46-0.56	0.56***	0.49-0.63	0.41***	0.39-0.44
No prior ASCVD, prior APT	0.87*	0.76-0.99	1.09	0.99-1.21	5.96***	5.34-6.66	3.63***	3.42-3.84
Prior ASCVD, prior APT	0.81***	0.73-0.90	1.07	0.98-1.17	6.17***	5.56-6.85	3.83***	3.62-4.06
**Discharge year (vs. 2009-11)**								
2012-14	1.24***	1.13-1.35	1.09*	1.00-1.17	1.27***	1.16-1.39	1.11***	1.05-1.16
2015-17	1.54***	1.39-1.71	1.27***	1.17-1.38	1.22***	1.11-1.36	1.27***	1.20-1.34

Note: (***): p<0.001; (**): p<0.01; (*): p<0.5; ASCVD: atherosclerotic cardiovascular disease; CI: Confidence Interval; MI: myocardial infarction; OR: Odds Ratio; PAD: peripheral arterial disease; [1] In the case of MI, stroke and PAD, the reference group is 'one comorbidity' as the Charlson Comorbidity Index classifies the presence of these conditions as a comorbidity.

Table I Association of patient characteristics with dual-antiplatelet therapy initiation (vs. monotherapy) among individuals with atherosclerotic cardiovascular disease who underwent percutaneous coronary intervention and initiated antiplatelet therapy, excluding individuals who did not initiate treatment (multivariable logistic regression model)

Percutaneous coronary intervention		
	OR	95% CI
**Female (vs. male)**	0.92	0.85-1.00
**Age (vs. 60-69 years old)**		
<50	1.22**	1.07-1.38
50-59 years	1.03	0.94-1.13
70-79 years	0.83***	0.75-0.91
80-80 years	0.82*	0.70-0.96
≥ 90 years	0.88	0.32-2.45
**Deprivation quintile (vs. 5, least deprived)**		
4	1.03	0.92-1.14
3	0.92	0.82-1.03
2	0.90	0.90-1.00
1 (most deprived)	0.82**	0.73-0.92
**Charlson Comorbidity Index (vs. no comorbidities)**		
1	1.71***	1.56-1.87
2	1.64***	1.48-1.83
3	1.43***	1.22-1.67
4 or more comorbidities	1.55***	1.26-1.91
**Receiving specialist mental health care**	1.22	0.80-1.86
**History of prior ASCVD or antiplatelet use (vs. no prior ASCVD, no prior antiplatelet)**		
Prior ASCVD, no prior APT	0.55***	0.47-0.64
No prior ASCVD, prior APT	0.15***	0.14-0.17
Prior ASCVD, prior APT	0.13***	0.12-0.15
**Discharge year (vs. 2009-11)**		
2012-14	1.33***	1.22-1.45
2015-17	1.63***	1.49-1.78

Note: (***): p<0.001; (**): p<0.01; (*): p<0.5; APT: antiplatelet therapy; ASCVD: atherosclerotic cardiovascular disease; CI = Confidence Interval; DAPT: dual-antiplatelet therapy; OR: Odds Ratio; PCI: percutaneous coronary intervention.

Table J Associations of patient characteristics with antiplatelet therapy discontinuation among individuals with atherosclerotic cardiovascular disease, by atherosclerotic cardiovascular disease type (multivariable Cox proportional hazards models)

	**MI**		**Ischaemic stroke**		**PAD**		**Other ASCVD**	
	HR	95% CI	HR	95% CI	HR	95% CI	HR	95% CI
**Female (vs. male)**	1.08***	1.03-1.14	1.01	0.96-1.07	0.99	0.91-1.07	1.16***	1.12-1.20
**Age (vs. 60-69 years old)**								
<50	1.10 *	1.02-1.19	1.21***	1.09-1.36	1.25*	1.05-1.49	1.22***	1.13-1.31
50-59 years	0.89**	0.83-0.96	0.92	0.83-1.01	1.01	0.88-1.15	0.96	0.91-1.01
70-79 years	1.35***	1.27-1.44	1.40***	1.30-1.51	1.27***	1.15-1.40	1.24***	1.19-1.30
80-80 years	1.90***	1.77-2.05	1.55***	1.42-1.68	1.74***	1.55-1.96	1.56***	1.47-1.66
≥ 90 years	2.87***	2.53-3.49	1.82***	1.57-.2.10	2.31***	1.74-3.06	1.91***	1.65-2.22
**Deprivation quintile (vs. 5, least deprived)**								
4	0.99	0.92-1.07	0.96	0.87-1.05	0.94	0.82-1.09	1.24***	1.13-1.36
3	1.03	0.96-1.11	0.89*	0.82-0.98	1.07	0.93-1.22	1.01	0.93-1.09
2	1.02	0.95-1.10	0.92	0.84-1.00	0.95	0.83-1.08	0.99	0.94-1.05
1 (most deprived)	1.03	0.96-1.11	0.87**	0.80-0.95	1.08	0.95-1.24	0.94**	0.90-0.98
**Charlson Comorbidity Index (vs. no comorbidities)**[10]								
1	N/A		N/A		N/A		0.94**	0.90-0.98
2	1.11***	1.05-1.17	1.01	0.95-1.08	1.01	0.91-1.12	0.99	0.94-1.05
3	1.33***	1.24-1.43	1.08	0.99-1.18	1.15*	1.01-1.31	1.01	0.93-1.09
4 or more comorbidities	1.66***	1.54-1.80	1.29***	1.16-1.42	1.40***	1.22-1.61	1.24***	1.13-1.36
**Receiving specialist mental health care**	1.77***	1.54-2.04	1.55***	1.34-1.80	1.50**	1.13-1.99	1.71***	1.53-1.91
**History of prior ASCVD or antiplatelet use (vs. no prior**								

[10] Absence of comorbidities as defined by CCI: in the case of the total ASCVD and other ASCVD populations, this means that individuals were not hospitalised for any of the 17 specified conditions within 12 months prior to and including the index admission. In the case of the MI, stroke and PAD populations, every individual has at least one CCI comorbidity, their index condition (i.e., MI, stroke or PAD), thus absence of comorbidity is not applicable (N/A).

	MI		Ischaemic stroke		PAD		Other ASCVD	
**ASCVD, no prior antiplatelet)**[11]								
Prior ASCVD, no prior APT	1.69***	1.57-1.83	1.30***	1.18-1.43	1.11	0.94-1.33	1.34***	1.24-1.46
No prior ASCVD, prior APT	1.12**	1.04-1.21	1.06	0.98-1.14	0.75***	0.67-0.84	0.76***	0.72-0.80
Prior ASCVD, prior APT	1.14***	1.07-1.21	1.12**	1.05-1.21	0.66***	0.59-0.73	0.70***	0.66-0.73
**Discharge year (vs. 2009-11)**								
2012-14	0.92**	0.88-0.97	1.05	0.99-1.12	0.98	0.90-1.07	0.99	0.95-1.03
2015-17	0.82***	0.76-0.88	0.85***	0.78-0.92	0.80**	0.71-0.91	0.79***	0.75-0.84

Note: (***): p<0.001; (**): p<0.01; (*): p<0.5; APT: antiplatelet therapy; ASCVD: atherosclerotic cardiovascular disease; CI: Confidence Interval; HR: Hazard Ratio; MI: myocardial infarction; PAD: peripheral arterial disease.[1]In the case of MI, stroke and PAD the reference group is ‘one comorbidity’ as the Charlson Comorbidity Index classifies the presence of these conditions as a comorbidity.

[11] For individuals with index hospitalisations between October 2009 and March 2010, information on prior medication use is available for the 6 to 11 months prior to index admission. For all discharges recorded after 1 April 2010, medication history is available for 12 months prior to index admission.

FIGURES

Figure A Overview of the linkage and study population

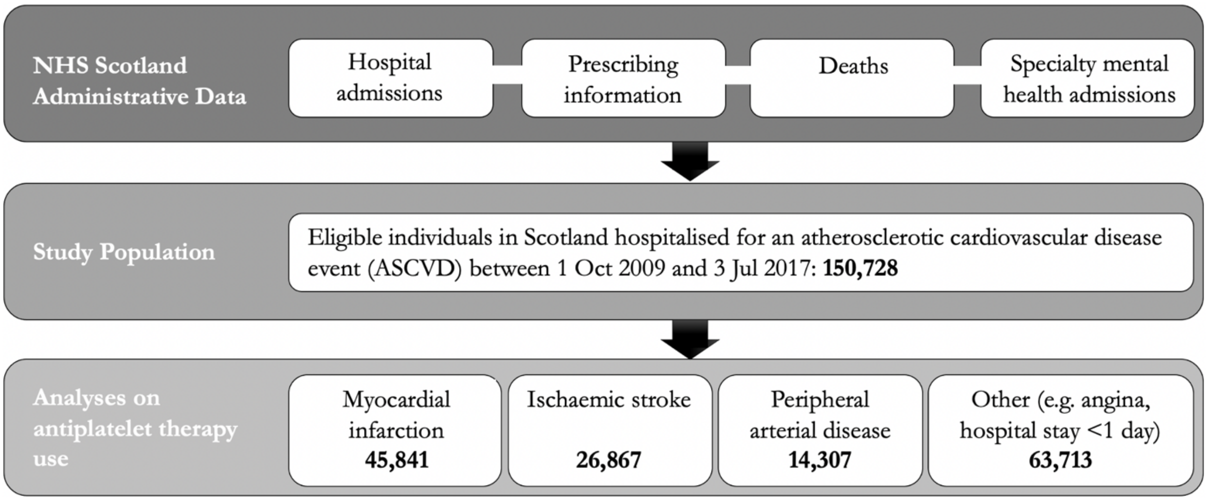

NHS: National Health Service

Figure B Flowchart of the study population

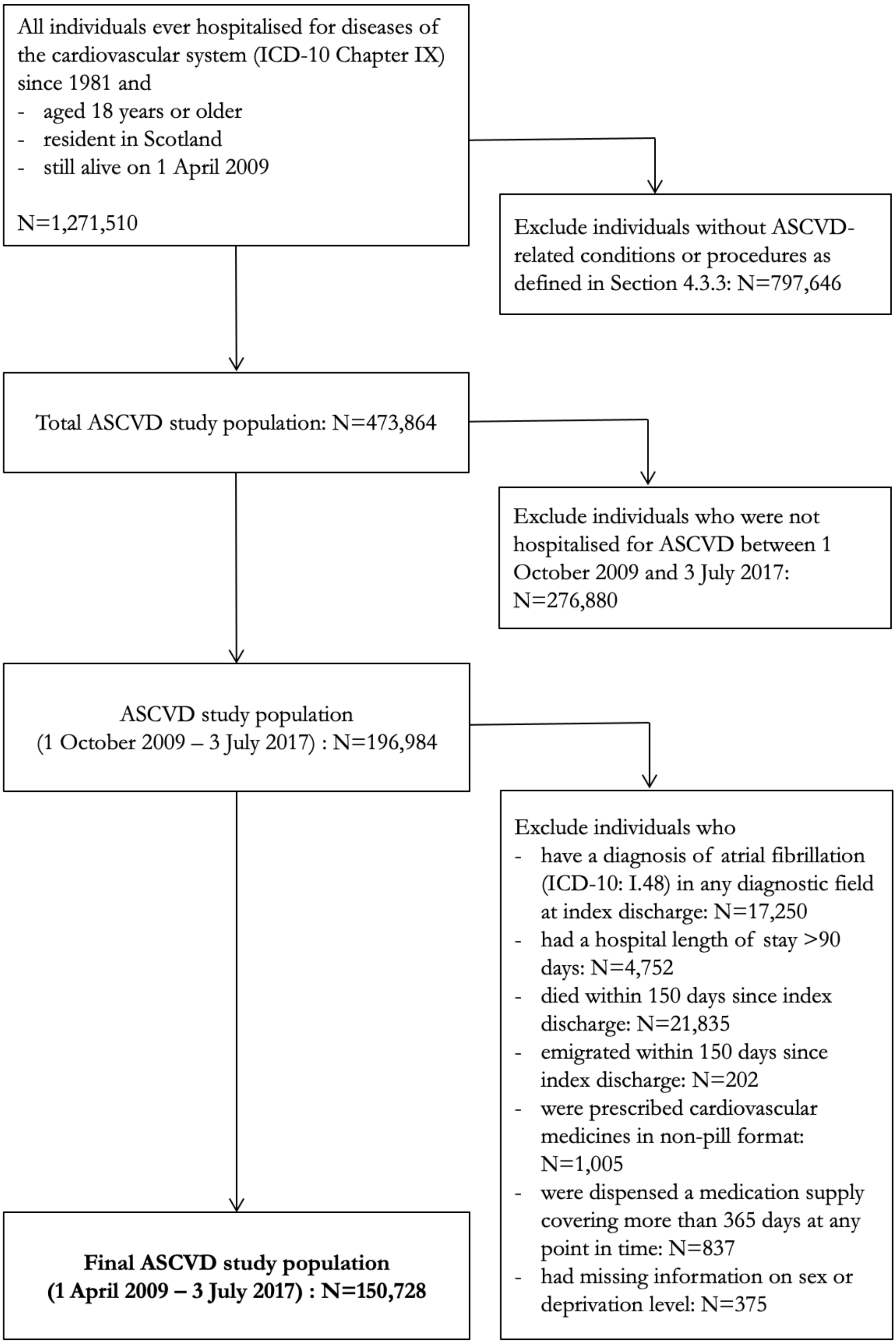

ASCVD: atherosclerotic cardiovascular disease; ICD-10: International Classification of Diseases Revision 10.

Figure C Antiplatelet therapy initiation and discontinuation rates over time following a myocardial infarction event

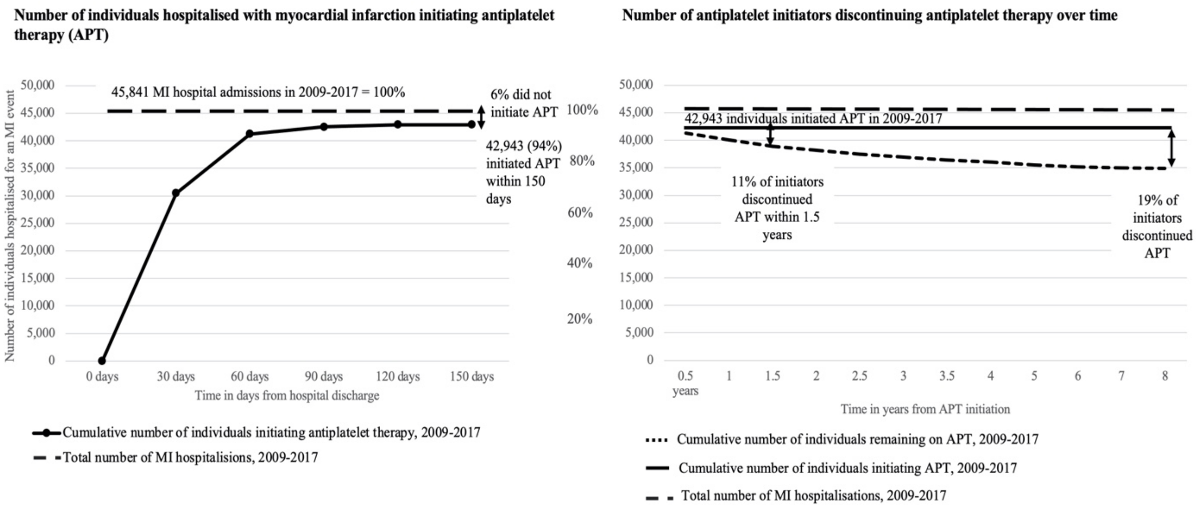

APT: antiplatelet therapy; MI: myocardial infarction

Figure D Antiplatelet therapy initiation and discontinuation rates over time following an ischaemic stroke event

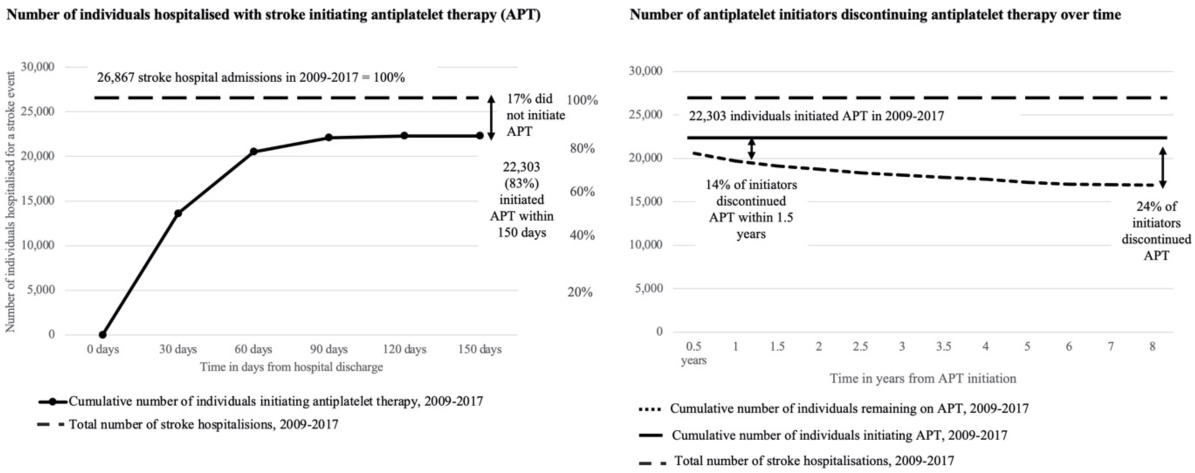

APT: antiplatelet therapy

Figure E Antiplatelet therapy initiation and discontinuation rates over time following a peripheral arterial disease event

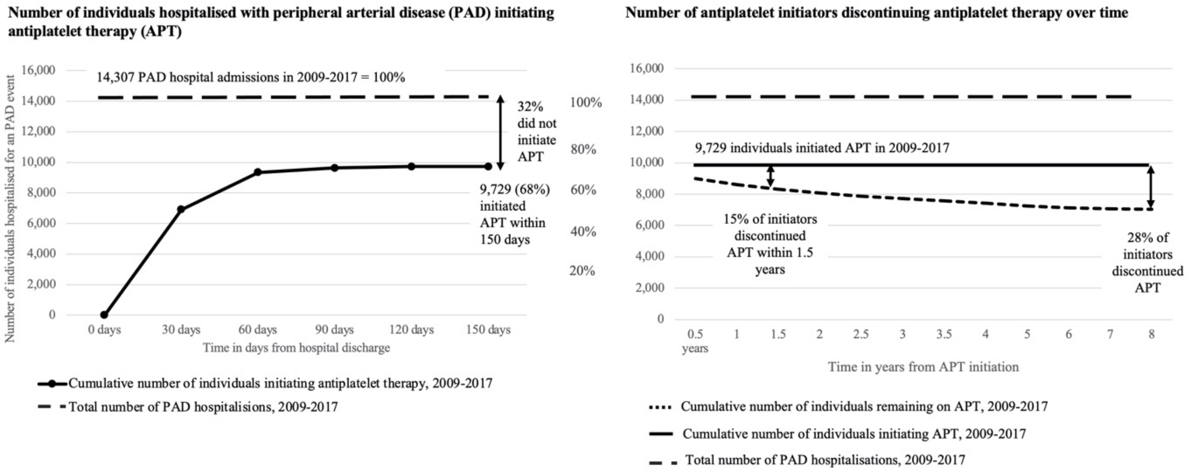

APT: antiplatelet therapy; PAD: peripheral arterial disease

Figure F Antiplatelet therapy initiation and discontinuation rates over time following other ASCVD events

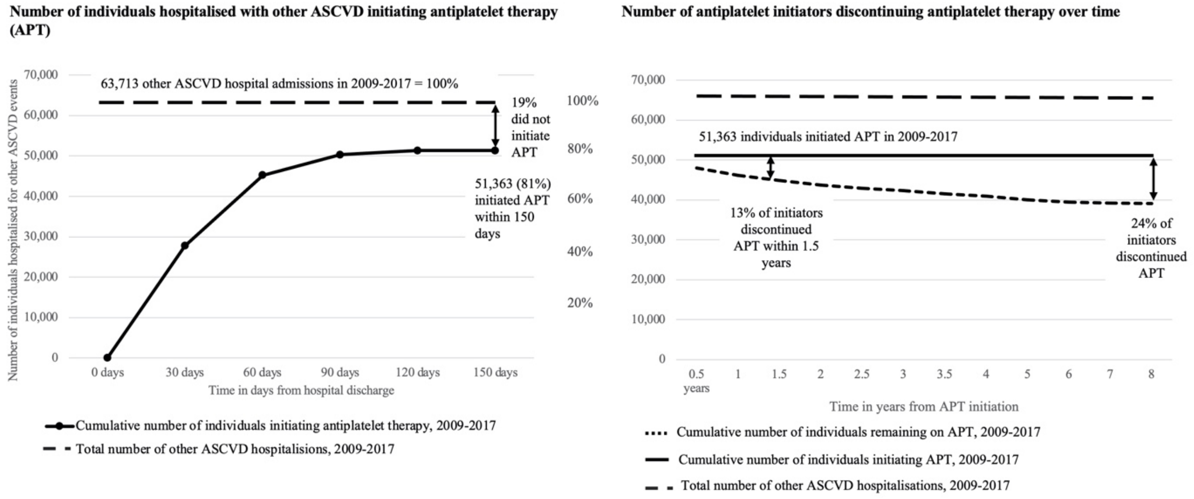

APT: antiplatelet therapy; ASCVD: atherosclerotic cardiovascular disease
